# Comparison of Quantum Transition Characteristics of Group II–VI (ZnO), Group III–V (GaN) Compound Semiconductors, and Intrinsic (Si) Semiconductors in Response to Externally Applied Energy

**DOI:** 10.3390/ma18204709

**Published:** 2025-10-14

**Authors:** Herie Park, Su-Ho Lee

**Affiliations:** 1 Department of Electrical Engineering, Dong-A University, Busan 49315, Republic of Korea; 2 Department of ICT Integrated Safe Ocean Smart Cities Engineering, Dong-A University, Busan 49315, Republic of Korea

**Keywords:** quantum transition characteristics, electron-phonon interaction, scattering coefficient, absorbed power

## Abstract

In this paper, we study the line-shape (LS), which indicates the amount of absorbed energy, and the line-width (LW), which indicates the scattering factor, according to the vibrational direction of the externally applied energy in the electron–phonon potential interaction system of representative semiconductor bonding types, group II–VI (ZnO) and group III–V (GaN) bonded compound semiconductors and pure group IV (Si) bonded semiconductors. One of the two systems receives the externally applied energy of right-handed circular polarization vibration, and the other receives the externally applied energy of left-handed circular polarization vibration. To analyze the quantum transport, we first employ quantum transport theory (QTR) for an electron system confined within a square-well potential, where the projected Liouville equation is addressed using the balanced-average projection method. In analyzing quantum transitions, phonon emission is linked to the transition line-width (LW), whereas phonon absorption is evaluated through the transition line-shape (LS), highlighting its sensitivity to temperature and magnetic field variations. As a result of analyzing the line-width (LW), which is a quantum scattering coefficient, and the line-shape (LS), which represents the absorbed power, the absorbed power and scattering coefficient were higher for the left circularly polarized vibration under the influence of the external magnetic field. In contrast, the right polarization produced smaller values. In addition, the scattering coefficient (LW) and the absorbed power according to the bonding type of the semiconductor were the largest in Si, a group IV bonded semiconductor, followed by group III–V (GaN) and group II–VI (ZnO) bonded semiconductors.

## 1. Introduction

As AI-related industries advance rapidly, semiconductors are increasingly required to possess diverse characteristics according to their functions. To meet these diverse demands, a deeper understanding of the quantum transition properties of semiconductor materials is essential. Therefore, this study comparatively analyzed the interactions between electron potential and phonons in ZnO, a representative group II–VI semiconductor with a typical bonding structure; GaN, a group III–V semiconductor; and Si, a pure group IV material. ZnO, with its excellent optical properties, is widely used in optoelectronics as devices and electrodes. Furthermore, its high electromechanical coupling coefficient has gained considerable attention for its applicability in piezoelectric devices. GaN, a next-generation semiconductor material, has recently attracted increasing attention as a high-speed, low-loss, and high-efficiency semiconductor stemming from its ability to surpass the physical limits of silicon and its high electron mobility, enabling high-speed switching and reduced power loss in systems [1,2]. Accordingly, so have the quantum transition properties of the two semiconductors with Si, which still serves as a representative semiconductor [3,4,5,6,7,8]. Therefore, in this study, we compared the interaction between the electron potential and phonons in accordance with the direction of the external input energy field in ZnO, GaN, and Si semiconductor materials [9,10,11]. The external input energy field is divided into two systems: one is an energy field applied with a right-handed circularly polarized field (RCF), while the second corresponds to an energy field applied with a left-handed circularly polarized field (LCF). This study is directed toward analyzing the line-shape (LS), which represents the degree of absorbed energy, and the line-width (LW), which reflects the scattering factor. In addition, the quantum transition characteristics were compared under two conditions. The first corresponds to a right-handed circularly polarized field, while the second involves an energy field. The analysis explores how these characteristics change with the direction of the applied field. There are numerous theories for quantum transport problems, including Boltzmann transport theory [12], Green’s function approach [13,14], the force balance approach [15], Feynman’s path integral formulation [16] and the projection operator techniques [17,18]. Despite the reasonableness of all these methods, research on nonlinear behavior has been restricted. A thorough view of electron transport in strong electric fields requires the inclusion of nonlinear effects. Furthermore, the method proposed by the research groups of Zwanzig and Kenkre directly employed the projection operator in the Liouville equation to propose a response function that incorporates Kubo’s theory as a lowest-order approximation [9,10,19,20]. The propagator includes a nonlinear factor, but since it appears in the exponent, extending this term is difficult.

Many methods have been proposed to interpret such quantum transport, and in this study, we employed quantum transport theory (QTR) for an electron system confined in a square-well potential. The present work applies the projected Liouville equation method with the Equilibrium Average Projection Technique (EAPT), which has the advantage of excellent extensibility of quantum transport theory and of being able to obtain the quantum response function as well as the scattering factor expression at once through this extension [11,21,22]. Previous studies have been limited to unbound potential systems using the extremely weak coupling (EWC) approximation and have also suffered from the lack of intermediate states in quantum transitions. However, the advantage of using the EAPS is that it extends quantum transport theory to obtain the quantum response function and scattering factor formulas in a single step. Regarding the dependence of the line-width (LW) on the magnetic field, which represents the scattering factor, it is difficult to calculate or measure the absorbed power at various external magnetic field wavelengths, making it very difficult to analyze using other theories or experiments. However, our research team proposed that the QTR of the EAPS can directly determine the line-width (LW) of the scattering factor at various external magnetic field wavelengths through the EAPS, making it easy to verify the variation in the line-width (LW) with magnetic field [23,24].

This study mainly aimed to investigate the transition characteristics of various semiconductor materials by comparing the line-shapes (LSs) of quantum-level optical transitions, which indicate the absorbed power according to the input of right circularly polarized fields (RCFs) and left circularly polarized fields (LCFs), with the quantum-level transition line-width (LW), corresponding to the scattering factor effect, in the electron-potential–phonon interaction system of representative semiconductor materials such as ZnO, GaN, and Si [24,25].

## 2. Expression for Absorbed Power and Scattering Factor

When the electron field is placed in a static magnetic field $\vec{B}=B_{z}\hat{z}$, the single electron energy level is quantized into Landau levels. Here, we choose an electron field confined within an infinite square well potential (SQWP) extending from z=0 and $z=L_{z}$ along a single direction, the z-direction. Also, the eigenvalues and eigenstates of the square well potential system are those in the reference [22]. Assuming that an electric field $E(t)=E_{0}\exp(i\omega t)$ is imposed along the z-axis, accompanied by displacement, the absorbed power appearing in the system is expressed as P(ω)=(E02/2)Re{σ(ω)}. Here, “Re” represents the real component, and σ(ω) is the photoconductivity tensor, representing the coefficient of the current expression. The absorbed power and the scattering factor represent the optical line-shape (LS) and the optical line-width (LW), respectively. The Hamiltonian describing the electron–phonon interaction is formulated as in Equation (1).

(1) $$H_{s}=H_{e}+H_{P}+V=\sum_{\beta }\langle \beta \left|h_{0}\right|\beta \rangle a_{\beta }^{+}a_{\beta }+\sum_{q}\hbar \omega_{q}b_{q}^{+}b_{q}+\sum_{q}\sum_{\alpha ,\mu }C_{\alpha ,\mu }\left(q\right)a_{q}^{+}a_{\mu }\left(b_{q}+b_{-q}^{+}\right)$$

$H_{e}$: Hamiltonian of an electron; $h_{0}$: Hamiltonian of a single-electron; $H_{P}$: Hamiltonian of a phonon; $b_{1}(b_{2}^{+})$: Boson particle annihilation operator (creation operator); V: interaction Hamiltonian of electron–phonon (or impurity); $\vec{q}$: wave vector of a phonon (or impurity). The electron–phonon (or impurity) interaction Hamiltonian of the interacting system is as follows:

(2) $$V\equiv \sum_{q}\sum_{\alpha ,\mu }C_{\alpha ,\mu }\left(q\right)a_{\alpha }^{+}a_{\mu }\left(b_{q}+b_{-q}^{+}\right)$$

here, the electron–phonon interaction coupling matrix element $C_{\alpha ,\mu }\left(q\right)$ is $C_{\alpha ,\mu }\left(q\right)\equiv V_{q}<\alpha |\exp(i\vec{q}\cdot \vec{r})|\mu >$, and here $V_{q}$ is the material’s coupling coefficient and $\vec{r}$ is the electron position vector. Using the successive approximation in the reference [23], the absorbed power formula in a right-circularly polarized external magnetic field (or LS formula) is finally obtained as Equation (3). We also proposed a specific absorbed power formula that constrains the potential system in the reference [24].

(3) $$P^{R}\left(\omega \right)\propto \left(\frac{e^{2}\omega_{c}^{2}}{\pi^{2}\hbar \omega }\right)\left\{\frac{\gamma_{total}^{R}\left(\omega_{c}\right)\sum_{N_{\alpha }}\int_{-\infty }^{\infty }dk_{z\alpha }\left(N_{\alpha }+1\right)\left(f_{\alpha }-f_{\alpha +1}\right)}{{\left(\omega -\omega_{c}\right)}^{2}+{\left(\gamma_{total}^{R}\left(\omega_{c}\right)\right)}^{2}}\right\}$$

Also, we can express the absorption power formula (or LS formula) for the energy input of the left-hand circular vibration external field as Equation (4).

(4) $$P^{\left(L\right)}\left(\omega \right)\propto \left(\frac{e^{2}\omega_{c}^{2}}{\pi^{2}\hbar \omega }\right)\left[\frac{\gamma_{total}^{\left(L\right)}\left(\omega_{c}\right)\sum_{N_{\alpha }}\int_{-\infty }^{\infty }dk_{z\alpha }\left(N_{\alpha }+1\right)\left(f_{\alpha +1}-f_{\alpha }\right)}{{\left(\omega -\omega_{c}\right)}^{2}+{\left(\gamma_{total}^{\left(L\right)}\left(\omega_{c}\right)\right)}^{2}}\right]$$

Here, the scattering factor function (or LW formula) for the energy applied to the right-hand circularly oscillating external field can be expressed as Equation (5).

(5) γtotal(R)ω≡ReΞkl(R)(ω)≡∑∓∑Nα=0∑Nβ=0γα,β∓=Ω4πℏ2υsπLz(2+δ(nα,nβ))∑∓∑Nα=0∑Nβ=0∫−∞∞dkzα∫−∞∞dqzYα,β(R)∓∑Nα=0∞∫−∞∞dkzαNα+1fα+1−fα

Also, the scattering factor function (or LW) for the energy applied to the left-hand circularly oscillating external field is given by Equation (6).

(6) γtotal(L)ω≡ReΞkl(L)(ω)≡∑∓∑Nα=0∑Nβ=0γα,β(L)∓=Ω4πℏ2υsπLz(2+δ(nα,nβ))∑∓∑Nα=0∑Nβ=0∫−∞∞dkzα∫−∞∞dqzYα,β(L)∓∑Nα=0∞∫−∞∞dkzαNα+1fα−fα+1

When a circularly polarized external magnetic field of various frequencies is applied in right- or left-handed directions, the dependence of LSs and LWs on the magnetic field and temperature for tetravalent-bonded semiconductor silicon (Si) and the group III–V and group II–VI bonded ones, gallium nitride (GaN) and zinc oxide (ZnO), were numerically analyzed using Equations (3)–(6). Since the absorbed power must be calculated for various frequencies, it is difficult to obtain the LWs using other theories for the analysis. However, the Equilibrium Average Projection Scheme (EAPS) allows for the direct calculation of the LWs, which facilitates the numerical analysis. By comparing the dependence of the LWs and LSs with the magnetic field and temperature, covering transitions both between and within Landau levels, the quantum transition process in germanium can be analyzed. The material constants of ZnO, GaN, and Si semiconductors are listed in Table 1. These constants were adopted for the numerical analysis. To compare LSs and LWs according to the direction of application of two polarized oscillating external magnetic fields, the left circular oscillating external magnetic field (LCF) is abbreviated as “L” in the graph, and the right circular oscillating external magnetic field (RCF) is abbreviated as “R”.

## 3. The Analysis

**Figure 1:**
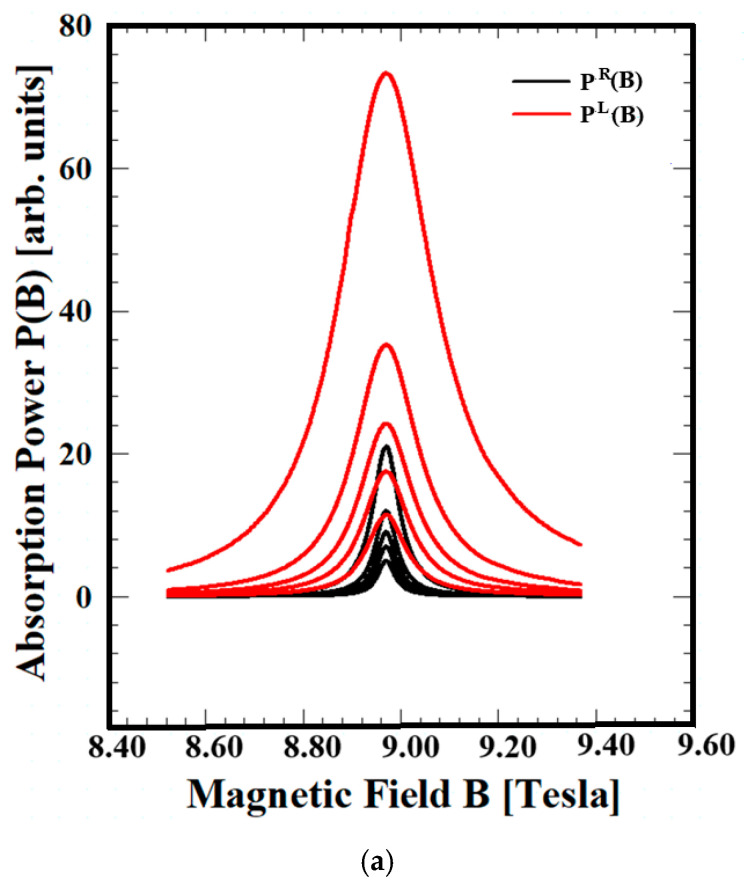

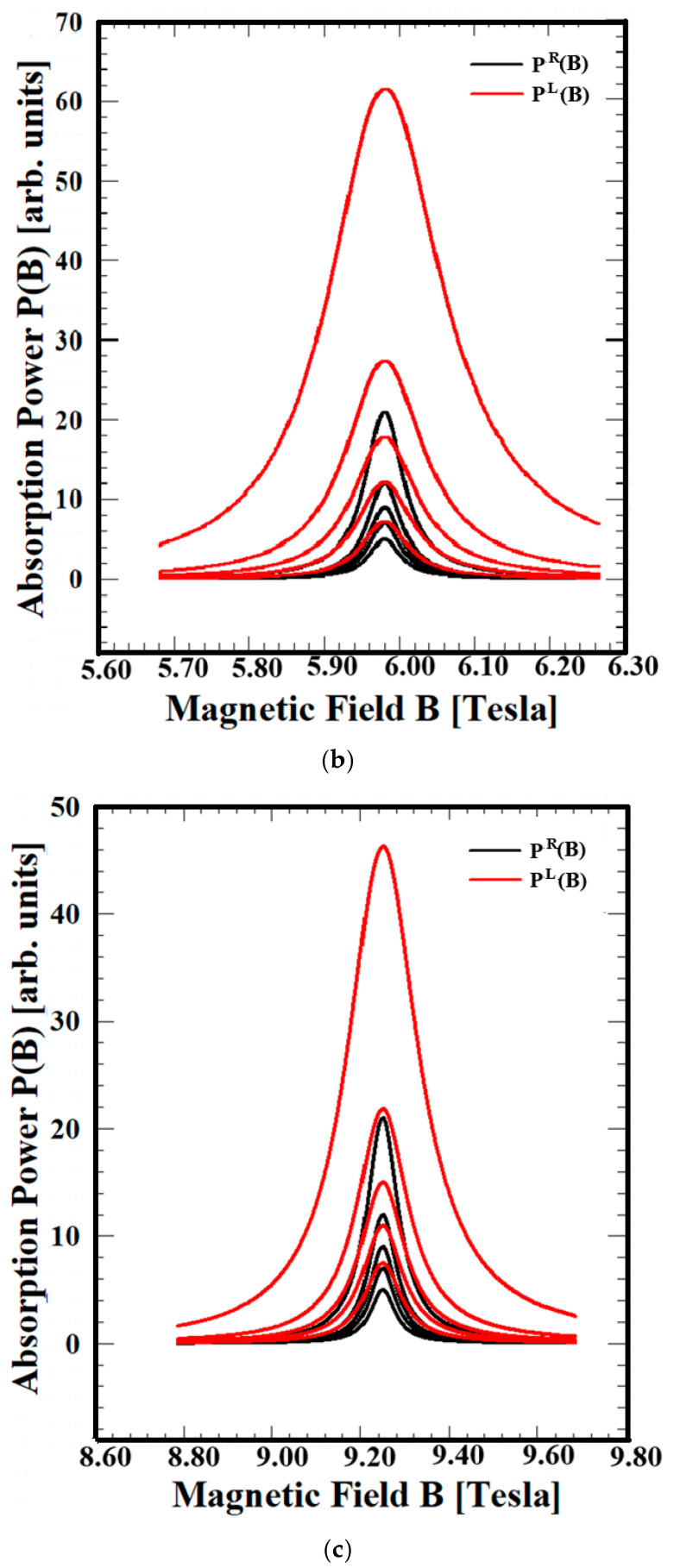
The magnetic field dependence of normalized  P(B) (LSs) of Si, GaN, and ZnO. (**a**) Magnetic field dependence of normalized  P(B) (LSs) of Si with  λ = 394 μm at T = 50, 70, 90, 120, and 210 K (ordered from bottom upward). (**b**) Magnetic field dependence of normalized  P(B) (LSs) of GaN with  λ = 394 μm at T = 50, 70, 90, 120, and 210 K (ordered from bottom upward). (**c**) Magnetic field dependence of normalized  P(B) (LSs) of ZnO with  λ = 394 μm at T = 50, 70, 90, 120, and 210 K (ordered from bottom upward).

Figure 1 compares the influence of the magnetic field on the absorption power P^R^(B) and P^L^(B) according to the line-shapes (LSs) of Si, GaN, and ZnO at temperatures of T = 50, 70, 90, 120, and 210 K, with an external magnetic field wavelength of λ = 394 μm. Figure 1 shows that the absorption power P(B) and the line-width (LW), which represents the scattering coefficient, increase with increasing temperature. These results indicate that the increase in lattice vibration due to the increase in heat increases the phonon collision effect, which can account for the resonance observed in the electron–phonon interaction system. The externally applied energy field is larger when applied by the LCF than when applied by the RCF. In addition, the absorption power P^R^(B) of all three materials is almost the same when applied by the LCF, but the P^L^(B) follows the order of Si > GaN > ZnO. This is because the difference in absorption power depends on the binding energy of the materials. These results demonstrate that the QTR of the EAPS effectively accounts for resonance phenomena, incorporating quantum transfer and scattering processes on a microscopic scale. The comparison of absorbed power P(B) in a magnetic field with a circular external field direction was first proposed by our research team. The QTR of EAPS is readily available compared to other theories, as P(B) in a circular external field is obtained by the QTR of the EAPS, which has a comparable theoretical framework.

**Figure 2:**
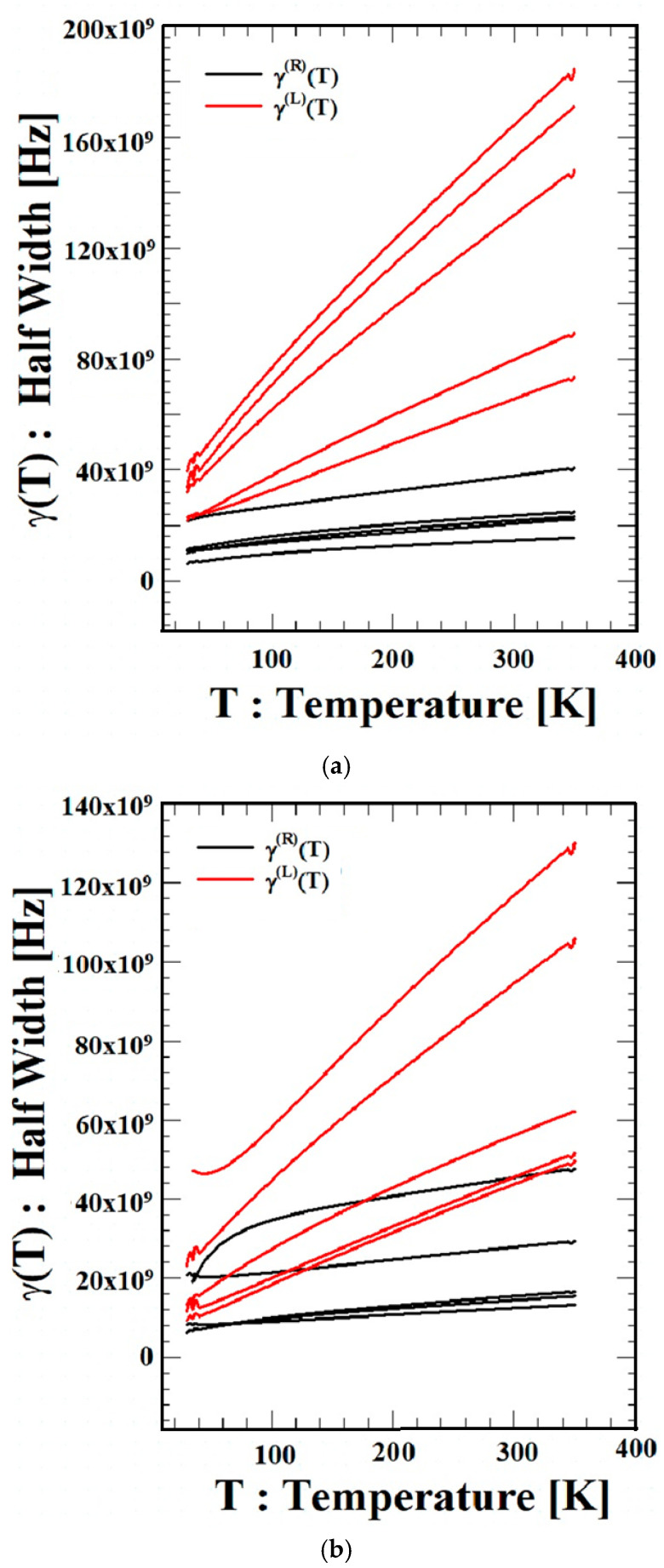

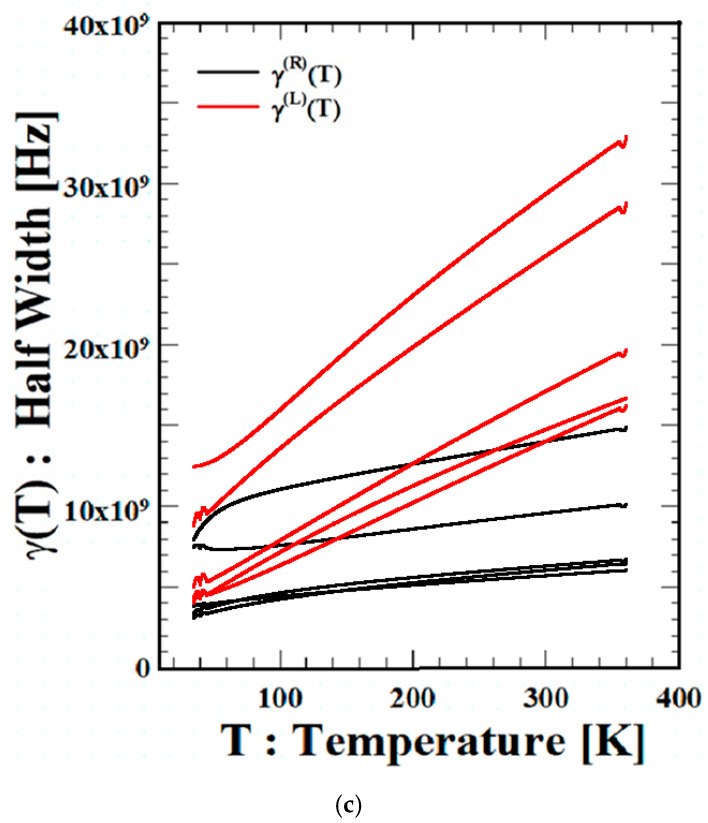
The temperature dependence of scattering factor (LWs),  γ(T), of Si, GaN, and ZnO. (**a**) Temperature dependence of QTLW of Si,  γ(T), with  λ = 220, 394, 513, 550, and 720 μm (ordered from bottom upward). (**b**) Temperature dependence of QTLW of GaN,  γ(T), with  λ = 220, 394, 513, 550, and 720 μm (ordered from bottom upward). (**c**) Temperature dependence of QTLW of ZnO,  γ(T), with  λ = 220, 394, 513, 550, and 720 μm (ordered from bottom upward).

Figure 2 shows the temperature dependences $\gamma^{\left(R\right)}(T)$ and $\gamma^{\left(L\right)}(T)$ of the scattering coefficient LWs. For all three materials (Si, GaN, and ZnO), $\gamma^{\left(L\right)}(T)$ increases with increasing temperature for external field wavelengths of  λ = 220, 394, 513, 550, and 720 μm, while $\gamma^{\left(R\right)}(T)$ also increases somewhat, but the change is not large. $\gamma^{\left(L\right)}(T)$ increases in the order of Si, a tetravalent semiconductor, GaN, a group III–V bond semiconductor, and ZnO, a group II–VI bond semiconductor. This finding agrees well with the results presented in Figure 1. In addition, $\gamma^{\left(L\right)}(T)$ increases with increasing temperature for all external field wavelengths, and $\gamma^{\left(L\right)}(T)$ is larger than $\gamma^{\left(R\right)}(T)$. This result indicates that the phonon scattering effect increases with increasing temperature for all external field wavelengths and that it is affected by the direction of electron motion. This can be seen as a result of increased energy absorption and emission due to increased thermal lattice vibration as temperature increases, as illustrated in Figure 1. Furthermore, it can be seen that the binding energy of the three semiconductors also affects the scattering coefficient.

**Figure 3:**
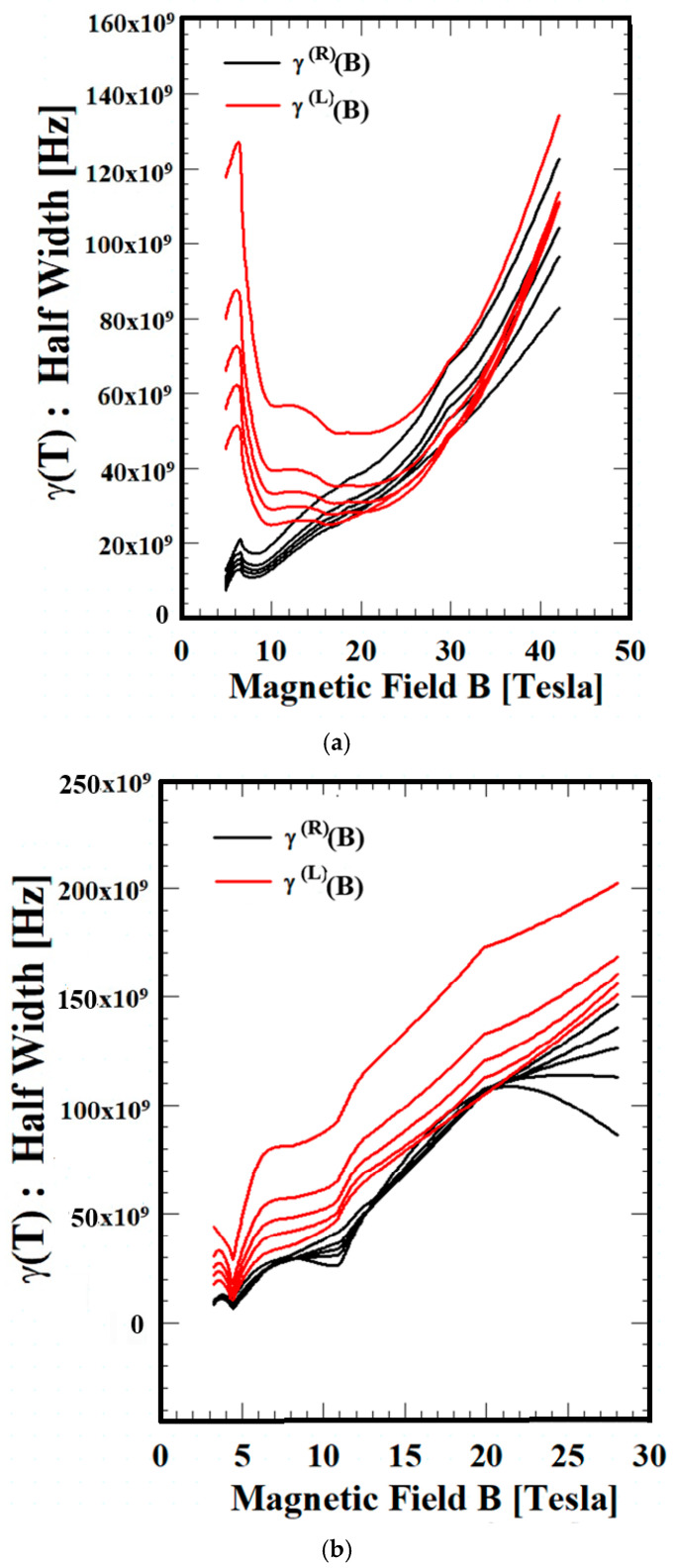

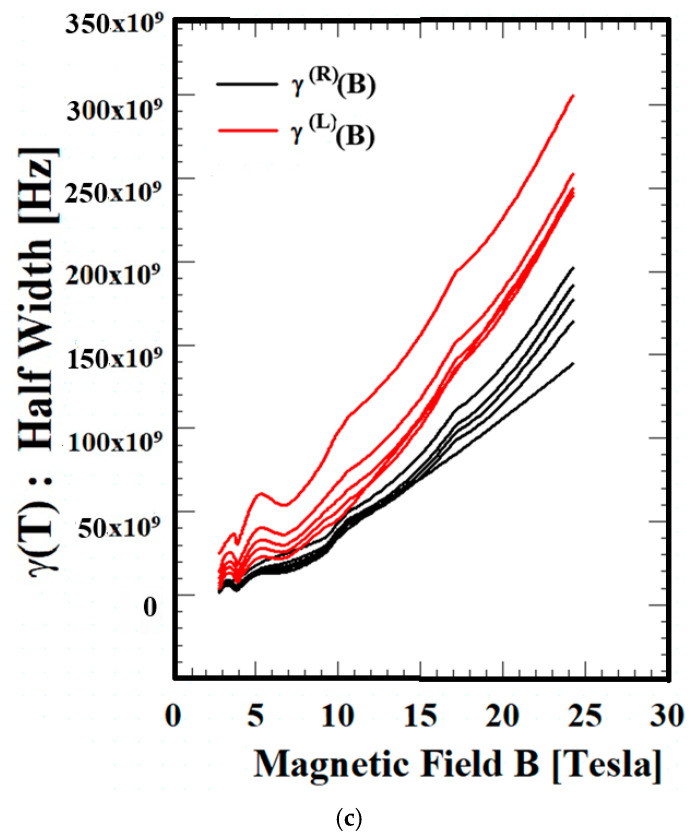
The magnetic field dependence of scattering factor (LWs),  γ(B), of Si, GaN, and ZnO. (**a**) Magnetic field dependence of LWs of Si,  γ(B), at T = 50, 70, 90, 120, and 210 K (ordered from bottom upward). (**b**) Magnetic field dependence of LWs of GaN,  γ(B), at T = 50, 70, 90, 120, and 210 K (ordered from bottom upward). (**c**) Magnetic field dependence of LWs of ZnO,  γ(B), at T = 50, 70, 90, 120, and 210 K (ordered from bottom upward).

Figure 3 shows the dependence of the magnetic field on the scattering factor LWs, $\gamma^{\left(R\right)}(B)$ and $\gamma^{\left(L\right)}(B)$, of three materials (Si, GaN, and ZnO) at T = 50, 70, 90, 120, and 210 K. For all three materials, $\gamma^{\left(L\right)}(B)$ is greater than $\gamma^{\left(R\right)}(B)$ under the full set of temperature conditions. This result also gives a consistent explanation of the directional characteristics of electron motion, depending on the magnetic field direction and system conditions. All three materials increase with increasing magnetic field, but for the Si semiconductor, the value decreases in the low-field region. Furthermore, for all three materials, the scattering factor LWs increase in the high-field region, with the values increasing in the order of ZnO > GaN > Si. Analyzing the scattering factor LWs at various wavelengths as a function of the magnetic field is very helpful in characterizing the magnetic properties of materials. Analyzing the magnetic field dependence at various wavelengths through theory or experimentation is difficult. This is because it is difficult to calculate or experimentally observe absorption and emission energies at various external magnetic field wavelengths. However, the QTR of the EAPS proposed in this study is an effective tool for directly measuring long-wavelength energy at various external magnetic field wavelengths.

For RFC, to investigate the process of the quantum transition, the total line-width (LW) is written as $\gamma_{total}^{\left(R\right)}\equiv \gamma_{em}^{\left(R\right)}+\gamma_{ab}^{\left(R\right)}$, where $\gamma_{em}^{\left(R\right)}\equiv \gamma_{intraL}^{\left(R\right)em}+\gamma_{interL}^{\left(R\right)em}$ and $\gamma_{ab}^{\left(R\right)}\equiv \gamma_{intraL}^{\left(R\right)ab}+\gamma_{interL}^{\left(R\right)ab}$ are the line-widths (LWs) of the overall phonon emission and absorption transitions, respectively. Here, $\gamma_{intraL}^{\left(R\right)em}$, $\gamma_{interL}^{\left(R\right)em}$,$\gamma_{intraL}^{\left(R\right)ab}$ and $\gamma_{interL}^{\left(R\right)ab}$ are the line-widths, and are defined, respectively, for the intra-level emission, inter-level emission, intra-level absorption, and inter-level emission-absorption transitions. For LFC, L is used instead of R in the subscript.

**Figure 4:**
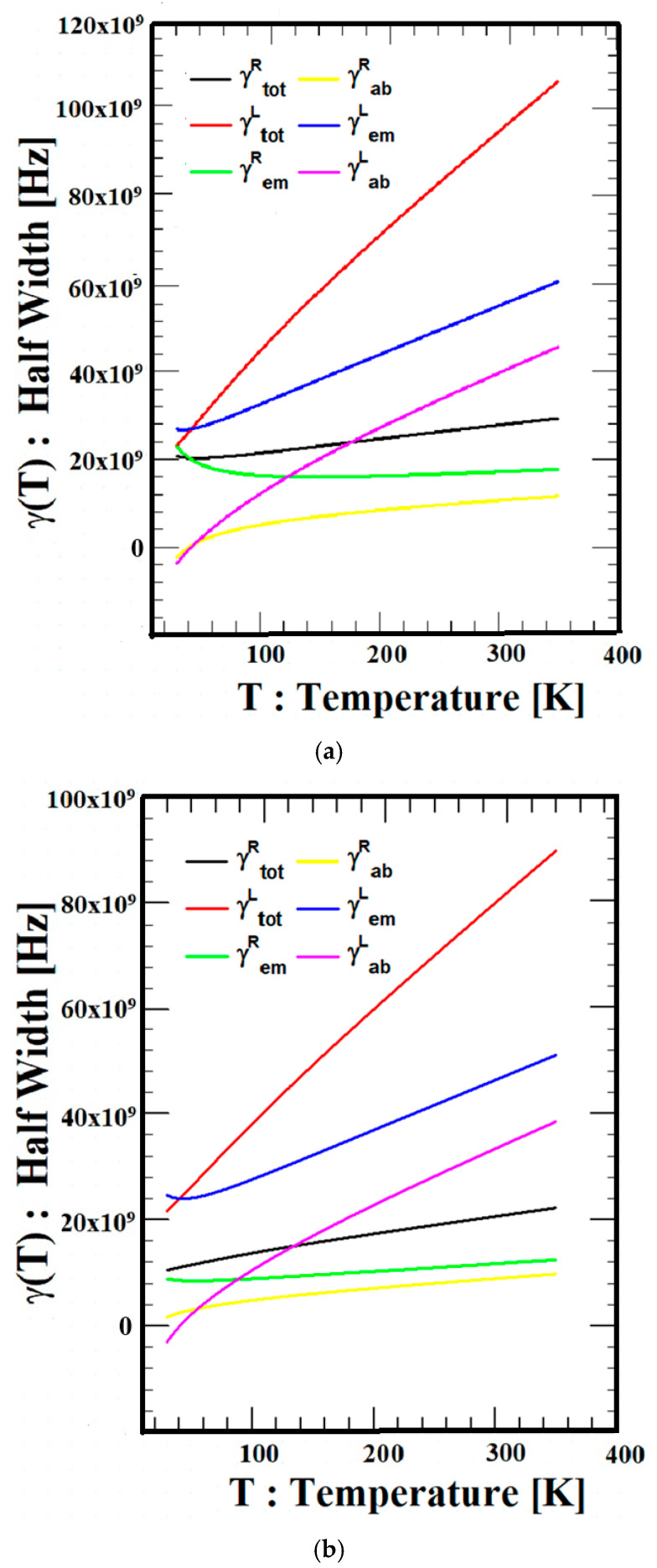

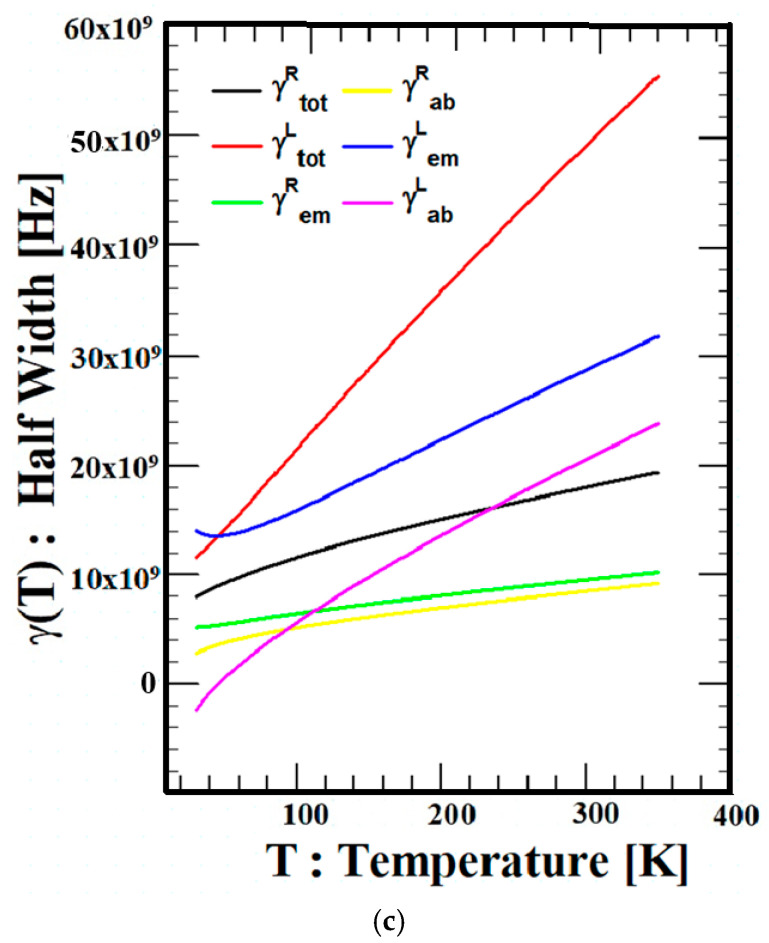
Temperature dependence of scattering factor (LWs),  γ(T), of Si, GaN, and ZnO. (**a**) Comparisons of the temperature dependence of LWs of Si, $\gamma_{total}(T)$, $\gamma_{em}(T)$, and $\gamma_{ab}(T)$ with  λ = 513 μm. (**b**) Comparisons of the temperature dependence of LWs of GaN, $\gamma_{total}(T)$, $\gamma_{em}(T)$, and $\gamma_{ab}(T)$ with  λ = 513 μm. (**c**) Comparisons of the temperature dependence of LWs of GaN, $\gamma_{total}(T)$, $\gamma_{em}(T)$, and $\gamma_{ab}(T)$ with  λ = 513 μm.

Figure 4 indicates the dependence of temperature on the line-widths (LWs), $\gamma_{total}^{\left(R\right)}(T)$, $\gamma_{em}^{\left(R\right)}(T)$, $\gamma_{ab}^{\left(R\right)}(T)$, $\gamma_{total}^{\left(L\right)}(T)$, $\gamma_{em}^{\left(L\right)}(T)$ and $\gamma_{ab}^{\left(L\right)}(T)$ of three types of semiconductors (Si, GaN, and ZnO) when the wavelength of the external applied magnetic field is  λ = 513 μm. The total line-width (LW) $\gamma_{total}^{\left(L\right)}(T)$, which is equal to the sum of phonon emission and absorption, increases with increasing temperature for all three materials because both phonon emission and absorption processes increase. In addition, $\gamma_{total}^{\left(R\right)}(T)$ does not increase much because both phonon emission and absorption processes show little change with temperature. In addition, the difference between RFC and LCF is regarded as the characteristics of the electron motion direction in accordance with the applied magnetic field direction and system conditions. Although the scattering behavior of transitions involving phonon emission and absorption cannot be experimentally separated, examining the relationship between the overall scattering behavior and the contributions of the two processes can provide insight into the thermal characteristics of the scattering effects in the system. The role of the two processes might differ across various systems and cases. In this study, the order of magnitudes of the left-hand line-widths (LWs) caused by the direction of the externally applied magnetic field is $\gamma_{ab}^{\left(L\right)}(T)$ < $\gamma_{em}^{\left(L\right)}(T)$ < $\gamma_{total}^{\left(L\right)}(T)$, and the order of magnitudes of the right-hand line-widths (LWs) is also $\gamma_{ab}^{\left(R\right)}(T)$ < $\gamma_{em}^{\left(R\right)}(T)$ < $\gamma_{total}^{\left(R\right)}(T)$ for all three semiconductors in the entire temperature range. Moreover, the analysis shows the value of the phonon emission transition, $\gamma_{em}^{\left(L\right)}(T)$ or $\gamma_{em}^{\left(R\right)}(T)$ is larger than the value of the phonon absorption transition, and $\gamma_{ab}^{\left(L\right)}(T)$ or $\gamma_{ab}^{\left(R\right)}(T)$ in all temperature ranges for all three semiconductors, regardless of the orientation of the externally applied magnetic field. These results help clarify the orientation-dependent behavior of the electron in relation to the applied magnetic field orientation and system conditions.

## 4. Practical Implications

By examining the behavior of electron–phonon interactions within quantum wells in response to external energy, we believe it will aid in the interpretation of conduction mechanisms in various semiconductor materials. Furthermore, by analyzing energy emission and absorption at specific wavelengths, we anticipate contributing to the development of optical devices that generate new optical wavelengths and optical sensors.

## 5. Conclusions

This study numerically analyzed the mechanism of scattering associated with electron–phonon coupling through strain potential in three types of semiconductors, a topic of recent research. Furthermore, we examined the electron dynamics relative to the orientation of the external magnetic field. First, the results of Equations (3)–(6) proposed in this study are more rigorous than those in previous studies and are directly applicable to numerical analysis via integration in the dual wave vectors. This investigation provides a distribution function that is significantly different with other approaches. Our EAPS theory, with its fewer computational steps, allows for a much easier analysis of quantum transitions in a variety of situations than other theories. Furthermore, the EAPS theory allows for the separation of line-widths for individual quantum transitions depending on the case. It facilitates the analysis of quantum transitions, which is a key advantage of the EAPS theory. The findings provide a basis for the numerical analysis of the scattering mechanism in materials with electron strain potential–phonon interaction. The characteristics of LWs and LSs increase with temperature and magnetic field for all three types of semiconductors. Si, a tetravalent semiconductor, showed the highest absorption power and scattering factor values, followed by GaN, a group III–V bond semiconductor, and ZnO, a group II–VI bond semiconductor. In addition, the absorption power as well as the scattering intensity increased under a leftward external magnetic field compared with the rightward orientation. In the scattering process, the scattering factor in the phonon emission transition process was larger than that in the transition process of phonon absorption, indicating that the phonon emission transition process is the main scattering process. This suggests that the EAPS theory can offer a promising means of elucidating material properties in other condensed matter systems.

## Figures and Tables

**Table 1:** Material constant.

Symbol	Contents	Value (ZnO)	Value (GaN)	Value (Si)
$m^{*}$	Effective mass of electron	0.24$m_{0}$	0.22$m_{0}$	0.33$m_{0}$
$\bar{m}$	Effective mass of hole	1.2$m_{0}$	0.58$m_{0}$	0.58$m_{0}$
ρ	Mass density	4090 kg/m^3^	4820 kg/m^3^	2340 kg/m^3^
κ	Characteristic constant	17.88 × 10^−4^ eV/K	8.58 × 10^−4^ eV/K	4.37 × 10^−4^ eV/K
ξ	Characteristic constant	204	235	636
$\bar{K}$	Electromechanical constant	6 × 10^−2^ m/s	2.6 × 10^−2^ m/s	2.98005 × 10^−2^ m/s
${\bar{v}}_{s}$	Speed of sound	4300.5 m/s	3045 m/s	9030 m/s
${\bar{\varepsilon }}_{s}$	Energy gap	3.4 eV	3.4 eV	1.424 eV
$L_{s}$ $L_{z}$	Length of well of z direction	20 × 10^−9^ m	20 × 10^−9^ m	20 × 10^−9^ m

## Data Availability

The original contributions presented in this study are included in the article. Further inquiries can be directed to the corresponding authors.
